# Protocol for low-input whole-genome bisulfite sequencing in *Arabidopsis*

**DOI:** 10.1016/j.xpro.2026.104928

**Published:** 2026-10-30

**Authors:** Jie Yang, Binh Thanh Vo, Frank Johannes

**Affiliations:** 1 Plant Epigenomics, TUM School of Life Sciences, Technical University of Munich, 85354 Freising, Germany

**Keywords:** Bioinformatics, Genomics, Plant sciences

## Abstract

DNA methylation profiling of rare plant samples is limited by the scarcity of input DNA. Here, we present a protocol for low-input whole-genome bisulfite sequencing (WGBS) in *Arabidopsis* in which DNA inputs as low as 50 pg can generate methylomes comparable to conventional WGBS. We describe steps for tissue cryomilling, Proteinase K lysis, and direct bisulfite conversion. We then detail procedures for post-bisulfite adapter tagging library construction and MethylStar-based alignment and methylation calling.

## Highlights

• Steps for plant tissue lysis and direct bisulfite conversion
• Instructions for random priming and post-bisulfite adapter ligation
• Steps for indexing PCR, library cleanup, and quality control before sequencing
• Read trimming, alignment, methylation calling, and imputation with MethylStar

## Abstract (editor highlights)

Publisher’s note: Undertaking any experimental protocol requires adherence to local institutional guidelines for laboratory safety and ethics.

## Graphical abstract

**Figure:**
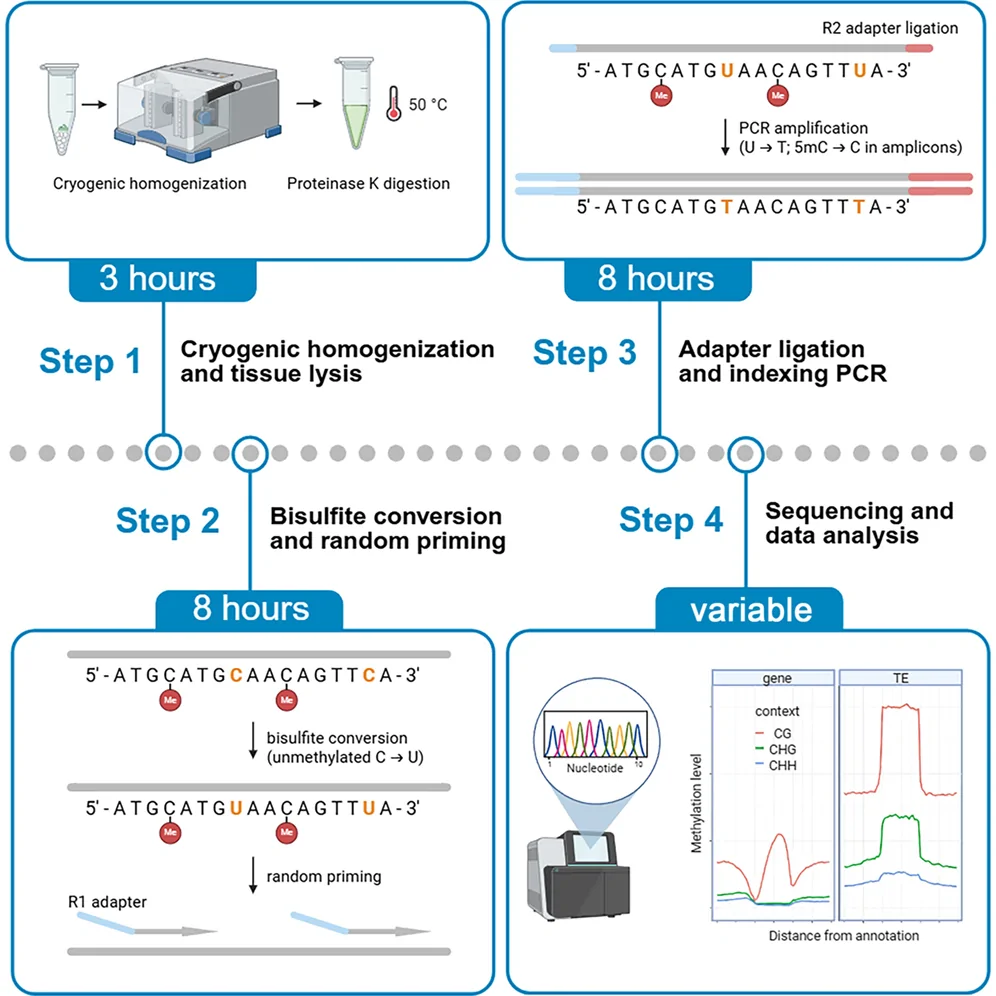

## Before you begin

This protocol describes a low-input WGBS workflow. Serially diluted *Arabidopsis* leaf DNA extracts were utilized as a quantitative benchmark to accurately characterize the appropriate DNA input range for this method. When processing actual rare specimens, no prior DNA extraction or isolation is required before entering this protocol.

We additionally validated the workflow using five independently dissected *Arabidopsis* shoot apical meristems (SAMs), with one SAM used directly for each library. Per library sequencing, alignment, genome coverage, and chloroplast conversion metrics are provided in Table S2. Applications to pollen, laser capture microdissected (LCM) tissues, and rare cell populations isolated via fluorescence activated cell sorting (FACS) were not systematically validated in this study and should be treated as unvalidated adaptations requiring specimen specific optimization.

### Innovation

Despite the growing demand for profiling trace samples in plant epigenomics, bulk WGBS protocols for ultra low DNA inputs (<200 pg) remain limited, particularly for tissues like the SAM, pollen, LCM tissues, and rare cell types isolated by FACS.

By utilizing the EZ DNA Methylation Direct Kit (Zymo Research, USA), this protocol performs bisulfite conversion directly from intact cells, thereby minimizing DNA loss associated with conventional extraction steps. The workflow can generate libraries suitable for genome wide CG methylation profiling from inputs equivalent to as little as 50 pg of DNA. To evaluate its performance and define a practical minimum input, we benchmarked the workflow against conventional bulk WGBS generated from 50 ng of DNA using a serial dilution of *Arabidopsis* thaliana leaf DNA ranging from 5 pg to 50 ng. At an input of 50 pg, the workflow produced robust sequencing results that were highly comparable to those obtained with conventional bulk WGBS. Although library construction was technically feasible with 5–10 pg of DNA, these lower inputs showed reduced library fidelity. We therefore recommend 50 pg as the routine minimum input.

### Institutional permissions

The *Arabidopsis thaliana* material used in this protocol does not require institutional permissions. Researchers applying this protocol to other organisms should confirm that all work conforms to the relevant institutional and national guidelines and obtain the necessary permissions before beginning.

### Plant material collection

**Timing: Variable**

1. Collect the target plant material.
  a. Collect tissues requiring cryogenic milling in 2 mL DNA LoBind tubes. For samples not requiring milling (e.g., FACS-sorted cells), use 0.2 mL PCR tubes.
  b. If tissue samples require storage before processing, flash-freeze them in liquid nitrogen.
  c. Store the frozen tissues at −80°C until further processing.

***Note:*** The recommended routine input is ≥50 pg DNA per reaction (approximately 150 diploid [2C] *Arabidopsis* cell equivalents). SAMs were used as a validation example, with one dissected SAM per library. SAMs were collected from 1-day-old seedlings grown under long-day conditions. Individual SAMs were dissected under a stereomicroscope using fine needles. Visible surrounding tissue was removed as completely as possible. DNA input was assessed separately from library preparation. DNA isolated from 3 individually dissected SAMs was quantified using the Qubit dsDNA HS Assay Kit. The measured amounts were 50.2, 62.4, and 70.8 pg per SAM (mean, 61.1 pg; n = 3).

## Key resources table

**Table:** 

REAGENT or RESOURCE	SOURCE	IDENTIFIER
**Biological samples**		

*Arabidopsis thaliana Col-0* leaf	This study	N/A
*Arabidopsis thaliana Col-0* SAM	This study	N/A

**Chemicals, peptides, and recombinant proteins**		

Klenow Fragment (3′ to 5′ exo-) (50 U/μL) & Blue Buffer (10×)	Enzymatics	Cat#P7010-HC-L
Exonuclease I (20 U/μL)	Enzymatics	Cat#X8010F
Shrimp Alkaline Phosphatase (rSAP)	New England BioLabs	Cat#M0371S or M0371L
AMPure® XP magnetic beads	Beckman Coulter	Cat#A63881
KAPA® HiFi HotStart ReadyMix	Roche	Cat#KK2602
dNTP Mix (10 mM each)	Thermo Fisher Scientific	Cat#18427013
Absolute ethanol (200-proof)	Sigma Aldrich	Cat#E7023
QIAquickTM Gel Extraction Kit	Qiagen	Cat#28704
Qubit™ dsDNA HS Assay Kit	Thermo Fisher Scientific	Cat#Q32851 or Q32854

**Critical commercial assays**		

EZ DNA Methylation Direct™ Kit	Zymo Research	Cat#D5020 or D5021
xGen™ Adaptase™ Module	IDT	Cat#10009826

**Oligonucleotides**		

P5L random primer (5 μM)	IDT	See sequences in materials and equipment
Custom P5L indexing primer (100 μM)	IDT	See sequences in materials and equipment
Custom P7L indexing primer (10 μM)	IDT	See sequences in materials and equipment

**Deposited data**		

Raw and processed WGBS data	This study	GEO: GSE338396
Arabidopsis thaliana TAIR10 reference genome (DNA FASTA)	Ensembl Plants/Ensembl Genomes, release 57	Assembly: GCA_000001735.1; https://ftp.ebi.ac.uk/ensemblgenomes/pub/release-57/plants/fasta/arabidopsis_thaliana/dna/Arabidopsis_thaliana.TAIR10.dna.toplevel.fa.gz
Arabidopsis thaliana TAIR10 genome annotation (GFF3)	Ensembl Plants/Ensembl Genomes, release 57	https://ftp.ebi.ac.uk/ensemblgenomes/pub/release-57/plants/gff3/arabidopsis_thaliana/Arabidopsis_thaliana.TAIR10.57.gff3.gz

**Software and algorithms**		

MethylStar (v1.5)	Shahryary et al.,^1^; this study	https://github.com/jlab-code/MethylStar; https://doi.org/10.5281/zenodo.23082324
Fastp	Chen, 2025, v1.3.3^2^	https://github.com/OpenGene/fastp
SAMtools	Li et al., 2009, v1.21^3^	http://samtools.sourceforge.net
Bowtie 2	Langmead & Salzberg, 2012, v2.5.4^4^	https://bowtie-bio.sourceforge.net/bowtie2/
Bismark	Krueger & Andrews, 2011, v0.24.2^5^	https://github.com/FelixKrueger/Bismark
METHimpute	Taudt et al., v1.28.0^6^	https://github.com/ataudt/methimpute
FastQC	Babraham Bioinformatics, v0.12.1	https://www.bioinformatics.babraham.ac.uk/projects/fastqc/

**Other**		

TissueLyser II	Qiagen	Cat#85300
NEBNext® Magnetic Separation Rack	New England Biolabs	Cat#S1515S
1.4mm Ceramic Bead Media	Omni	Cat#19-645-3
DNA LoBind Tubes 2.0 mL	Eppendorf	Cat#0030108426
PCR Tube Strips 0.2 mL	Eppendorf	Cat#951010022
high sensitivity Bioanalyzer® chip	Agilent	Cat#5067-4626

## Materials and equipment

**Table:** Proteinase K digestion mix

Reagent	Volume per reaction (μL)
M-Digestion Buffer (2×)	13
Proteinase K	1
Nuclease free H_2_O	12
**Total**	**26**

**Note:** M-Digestion Buffer (2×) and Proteinase K are supplied with the EZ DNA Methylation Direct Kit.

Prepare fresh immediately before use; keep on ice and use within 1 h. Do not store.

**Table:** Random priming master mix

Reagent	Volume per reaction (μL)
Blue Buffer (10×)	2
Klenow exo- (50 U/μL)	1
dNTPs (10 mM each)	1
Nuclease free H_2_O	6
**Total**	****10****

Prepare fresh immediately before use; keep on ice and use within 1 h. Do not store.

**Table:** Adaptase master mix

Reagent	Volume per reaction (μL)
Low EDTA TE	4.25
Buffer G1	2.0
Reagent G2	2.0
Reagent G3	1.25
Enzyme G4	0.5
Enzyme G5	0.5
**Total**	**10.5**

**Note:** Buffer G1, Reagent G2, Reagent G3, Enzyme G4, and Enzyme G5 are supplied with the xGen™ Adaptase™ Module.

Prepare fresh immediately before use; keep on ice and use within 1 h. Do not store. Add to the DNA only after denaturation.

**Table:** Indexing PCR mix

Reagent	Volume per reaction (μL)
Custom P5L indexing primer (100 μM)	0.3
Custom P7L indexing primer (10 μM)	5
KAPA HiFi HotStart ReadyMix (2×)	25
**Total**	**30.3**

Prepare fresh immediately before use; keep on ice and use within 1 h. Do not store.

### Oligonucleotide sequences

The oligonucleotides used for library preparation are listed below.

### P5L random primer

Sequence (5′-3′):

/5SpC3/TTCCCTACACGACGCTCTTCCGATCT(N1:25252525) (N1) (N1) (N1) (N1) (N1) (N1) (N1) (N1).

Modification:/5SpC3/denotes a 5′C3 spacer modification, and each N1 specifies an equal mixture of A, C, G, and T.

Working solution: Dilute the P5L random primer to 5 μM with Low EDTA TE supplied with the xGen™ Adaptase™ Module.

### Indexing primers

The i5 and i7 indexing primers used for library amplification are listed below. The index sequence within each primer is shown in bold.

Working solutions: Prepare the i5 indexing primers at 100 μM, and the i7 indexing primers at 10 μM using Low EDTA TE supplied with the xGen™ Adaptase™ Module.

**Table:** i5 indexing primers

Name	Sequence (5′ to 3′)
P5L_D501	AATGATACGGCGACCACCGAGATCTA CAC**TATAGCCT**ACACTCTTTCCCTACA CGACGCTCT
P5L_D502	AATGATACGGCGACCACCGAGATCTA CAC**ATAGAGGC**ACACTCTTTCCCTACA CGACGCTCT
P5L_D503	AATGATACGGCGACCACCGAGATCTA CAC**CCTATCCT**ACACTCTTTCCCTACA CGACGCTCT
P5L_D504	AATGATACGGCGACCACCGAGATCTA CAC**GGCTCTGA**ACACTCTTTCCCTAC ACGACGCTCT
P5L_D505	AATGATACGGCGACCACCGAGATCTA CAC**AGGCGAAG**ACACTCTTTCCCTAC ACGACGCTCT
P5L_D506	AATGATACGGCGACCACCGAGATCTA CAC**TAATCTTA**ACACTCTTTCCCTACAC GACGCTCT
P5L_D507	AATGATACGGCGACCACCGAGATCTA CAC**CAGGACGT**ACACTCTTTCCCTACA CGACGCTCT
P5L_D508	AATGATACGGCGACCACCGAGATCTA CAC**GTACTGAC**ACACTCTTTCCCTACACG ACGCTCT

**Table:** i7 indexing primers

Name	Sequence (5′ to 3′)
P7L_D701	CAAGCAGAAGACGGCATACGAGAT **CGAGTAAT**GTGACTGGAGTTCAGA CGTGTGCTCTT
P7L_D702	CAAGCAGAAGACGGCATACGAGAT **TCTCCGGA**GTGACTGGAGTTCAGA CGTGTGCTCTT
P7L_D703	CAAGCAGAAGACGGCATACGAGAT **AATGAGCG**GTGACTGGAGTTCAGA CGTGTGCTCTT
P7L_D704	CAAGCAGAAGACGGCATACGAGAT **GGAATCTC**GTGACTGGAGTTCAGAC GTGTGCTCTT
P7L_D705	CAAGCAGAAGACGGCATACGAGAT **TTCTGAAT**GTGACTGGAGTTCAGAC GTGTGCTCTT
P7L_D706	CAAGCAGAAGACGGCATACGAGAT **ACGAATTC**GTGACTGGAGTTCAGA CGTGTGCTCTT
P7L_D707	CAAGCAGAAGACGGCATACGAGAT **AGCTTCAG**GTGACTGGAGTTCAGA CGTGTGCTCTT
P7L_D708	CAAGCAGAAGACGGCATACGAGAT **GCGCATTA**GTGACTGGAGTTCAGAC GTGTGCTCTT
P7L_D709	CAAGCAGAAGACGGCATACGAGAT **CATAGCCG**GTGACTGGAGTTCAGA CGTGTGCTCTT
P7L_D710	CAAGCAGAAGACGGCATACGAGAT **TTCGCGGA**GTGACTGGAGTTCAGA CGTGTGCTCTT
P7L_D711	CAAGCAGAAGACGGCATACGAGAT **GCGCGAGA**GTGACTGGAGTTCAGA CGTGTGCTCTT
P7L_D712	CAAGCAGAAGACGGCATACGAGAT **CTATCGCT**GTGACTGGAGTTCAGAC GTGTGCTCTT

## Step-by-step method details

***Note:*** Fresh or flash-frozen tissues enter the workflow at step 1, isolated cells and LCM samples enter at step 2, and purified DNA at step 3. For FFPE samples, remove paraffin before starting at step 2. This workflow was not validated for LCM or FFPE samples in this study.

**CRITICAL:** Use nuclease-free tubes throughout. Use 2.0 mL DNA LoBind tubes for sample handling and PCR-clean 0.2 mL tube strips for reactions and thermal cycling (see key resources table).

### Tissue disruption

**Timing: 3–7 h, depending on the Proteinase K digestion step**

This step homogenizes flash frozen tissue and digests it with Proteinase K to release intact, bisulfite accessible chromatin.

1. Cryo milling.
  a. Pre chill the TissueLyser adapter blocks and 1.4 mm beads at −80°C or in liquid nitrogen before use.
  b. Add pre-chilled 1.4 mm beads to the 2 mL DNA LoBind tube containing the sample.
  c. Homogenize the samples using a TissueLyser II or a comparable homogenizer at 24 Hz for 2 min.
  d. Rotate the adapter so that the side closest to the instrument body becomes the furthest side.
  e. Homogenize for another 2 min to ensure even disruption.
2. Tissue lysis.
  a. Prepare Proteinase K, CT Conversion Reagent, and M-Wash Buffer according to the manufacturer’s instructions for the EZ DNA Methylation Direct Kit.
  b. Prepare the **Proteinase K digestion mix** (see materials and equipment).
  c. Add 26 μL Proteinase K digestion mix to each sample, mix thoroughly, and pulse-spin.
  d. Incubate at 50°C for 20 min. For LCM and FFPE samples, extend the incubation time to 4 h.
  e. After digestion, centrifuge at 10,000 × g for 5 min.
  f. Transfer 20 μL of the supernatant to a fresh PCR tube for bisulfite conversion, avoiding insoluble material.

***Note:*** The use of LCM and FFPE samples represents an unvalidated adaptation of this protocol and may require specimen specific optimization.

### Bisulfite conversion

**Timing: 5 h**

This step deaminates unmethylated cytosines to uracil.

3. Prepare the bisulfite conversion reaction.
  a. Use 20 μL of the tissue lysate supernatant from step 2. For purified DNA, adjust the sample volume to 20 μL with nuclease-free water.
  b. Add 130 μL of prepared CT Conversion Reagent to the PCR tube containing 20 μL of sample.
  c. Mix thoroughly by pipetting up and down and pulse-spin to collect liquid from the cap and tube walls.
4. Proceed with the bisulfite conversion of the DNA.
  a. Place the samples in a thermal cycler with the heated lid set to 105°C and run the following program:

**Table:** 

Temperature	Duration
98°C	8 min
64°C	3.5 h
4°C	hold, up to 20 h

**Pause point**: After bisulfite conversion, samples can be held at 4°C for up to 20 h before proceeding to step 5.

5. Bind the DNA.
  a. Place the Zymo-Spin IC Column into the provided collection tube.
  b. Add 600 μL M-Binding Buffer to each column.
  c. Transfer the bisulfite converted sample from step 4 into the column containing M-Binding Buffer.
  d. Close the cap and invert the column several times to mix.
  e. Centrifuge at >10,000 × *g* for 30 s.
  f. Discard the flow through.
6. Wash the column (wash 1).
  a. Add 100 μL M-Wash Buffer to the column.
  b. Centrifuge at >10,000 × *g* for 30 s.
7. Desulphonate the DNA.
  a. Add 200 μL M-Desulphonation Buffer to the column.
  b. Incubate at 20°C–30°C for 15–20 min.
  c. Centrifuge at >10,000 × *g* for 30 s.
8. Wash the column (wash 2).
  a. Add 200 μL M-Wash Buffer to the column and centrifuge at >10,000 × *g* for 30 s.
  b. Add another 200 μL M-Wash Buffer and centrifuge again for 30 s.
9. Elute the DNA.
  a. Place the Zymo-Spin IC Column into a clean 1.5 mL microcentrifuge tube.
  b. Add 12 μL low EDTA TE Buffer (provided with the xGen Adaptase Module) directly to the column matrix.
  c. Centrifuge at >10,000 × *g* for 30 s to elute the DNA.
  d. Store at −20°C if the samples are not processed immediately.

***Note:*** Use 9 μL bisulfite converted DNA for the subsequent random priming step.

### Random priming

**Timing: 2 h**

This step denatures bisulfite converted single stranded DNA, anneals a tagged random primer, and extends the primer with Klenow exo- to synthesize the first strand. Excess primers are then removed enzymatically with Exonuclease I and rSAP before downstream adapter ligation.

10. Denature the bisulfite converted DNA.
  a. Transfer 9 μL bisulfite converted DNA to a PCR clean 0.2 mL PCR tube.
  b. Incubate at 95°C for 3 min in a thermal cycler with the heated lid set to 105°C.
  c. Immediately transfer the samples to ice for approximately 2 min.
11. Add the random primer.
  a. Pulse-spin the samples.
  b. Add 1 μL 5 μM P5L random primer to each tube.
  c. Vortex gently and pulse-spin again to collect the liquid at the bottom of the tube.
12. Prepare the random priming master mix.
  a. Prepare the **random priming master mix** on ice (see materials and equipment).
  b. Keep the master mix on ice until use.

**CRITICAL:** Do not add the random priming master mix to the DNA primer mixture at this step.

13. Denature the DNA primer mixture.
  a. Incubate the DNA primer mixture at 95°C for 3 min in a thermal cycler with the heated lid set to approximately 105°C.
  b. Immediately transfer the samples to ice for 2 min.
14. Add the random priming master mix.
  a. Pulse-spin the samples.
  b. Add 10 μL random priming master mix to each tube.
  c. Vortex gently and pulse-spin again.
15. Run the random priming reaction.
  a. Place the samples in a thermal cycler and run the following program:

**Table:** 

Temperature	Duration
4°C	5 min, then ramp to 25°C at 0.1°C/s
25°C	5 min, then ramp to 37°C at 0.1°C/s
37°C	60 min
4°C	hold

16. Remove excess primers and dNTPs enzymatically.
  ***Note:*** Residual single stranded primers are digested with Exonuclease I, while rSAP dephosphorylates unincorporated dNTPs before bead cleanup and Adaptase ligation.
  a. Pulse-spin the samples.
  b. Add 2 μL Exonuclease I (20 U/μL) and 1 μL Shrimp Alkaline Phosphatase (1 U/μL) directly to each completed random priming reaction.
  c. Mix gently and pulse-spin.
  d. Incubate the samples in a thermal cycler and run the following program:

**Table:** 

Temperature	Duration
37°C	30 min
4°C	hold

### AMPure magnetic bead cleanup

**Timing: 20–40 min, plus 30 min for bead equilibration**

This step removes enzymes and primers before adapter ligation.

17. Clean up the reaction with AMPure beads.
  a. Equilibrate the AMPure beads at 20°C–30°C for 30 min before use.
  b. Vortex the beads until fully resuspended.
  c. Add 18.4 μL AMPure beads (0.8× sample volume) to each tube.
  d. Mix thoroughly by gentle vortexing and pulse-spin to collect the liquid.
  e. Incubate for 5 min at 20°C–30°C.
  f. Place the tubes on a magnetic rack for 5 min.
  g. Carefully discard the supernatant.
  h. Wash the beads twice with 200 μL of freshly prepared 80% ethanol per wash, keeping the tubes on the magnetic rack.
  i. After the second ethanol wash, remove residual ethanol and air dry the beads for less than 5 min without overdrying.
  j. Add 11 μL low EDTA TE Buffer.
  k. Incubate for 5 min at 20°C–30°C.
  l. Place the tubes on the magnetic rack for 5 min.
  m. Transfer 10 μL clear supernatant to a fresh PCR tube.

**CRITICAL:** Do not overdry the beads, as this can significantly reduce DNA yield. See troubleshooting**,**problem 1.

### Adaptase ligation

**Timing: 1 h**

This step ligates a single stranded adapter to the 3′ end of the tagged fragments using the xGen Adaptase Module.

18. Prepare the Adaptase master mix.
  a. Prepare the **Adaptase master mix** on ice (see materials and equipment).
  b. Keep the master mix on ice until use.

**CRITICAL:** Do not add the Adaptase master mix to the DNA at this step.

19. Denature the DNA.
  a. Place 10 μL purified DNA in a thermal cycler at 95°C for 3 min with the heated lid set to 105°C.
  b. Immediately transfer the samples to ice and incubate for 2 min.
20. Start the Adaptase reaction immediately after denaturation.
  a. Pulse-spin the denatured DNA samples.
  b. Immediately add 10.5 μL Adaptase master mix to each tube.
  c. Vortex gently and pulse-spin.
  d. Place the samples in a thermal cycler with the heated lid set to 105°C and run the following program:

**Table:** 

Temperature	Duration
37°C	30 min
95°C	2 min
4°C	hold

### Optional QC checkpoint

**Timing: 3 h**

This optional pilot PCR can be used when establishing the protocol for a new sample type or when the optimal amplification cycle number is uncertain. It provides an early assessment of library formation and offers guidance on the number of cycles required for the main library amplification.

21. Perform a pilot PCR.
  a. Transfer 5.1 μL (one quarter) of the approximately 20.5 μL Adaptase tagged library to a new PCR tube.
  b. Add 30.3 μL **indexing PCR mix** (see materials and equipment).
  c. Add 15.4 μL nuclease free H_2_O to bring the total reaction volume to approximately 50.8 μL.
  d. Amplify the pilot reaction for 21 cycles using the cycling conditions described below.

**Table:** 

Temperature	Duration	Cycles
95°C	2 min	1
98°C	30 s	21
64°C	15 s	
72°C	30 s	
72°C	5 min	1
4°C	hold	–
  e. Add 40.6 μL AMPure beads (0.8× the 50.8 μL PCR product volume).
  f. Continue the cleanup as described in steps 17d-m.
  g. Measure the DNA concentration using Qubit and assess the fragment size distribution using a Bioanalyzer.
    ***Note:*** Use the pilot PCR yield to determine the number of cycles for amplification of the remaining three quarter template. Determine the main PCR cycle number from the pilot yield, accounting for the 3 fold larger template input. See troubleshooting, problem 1.

### Indexing PCR

**Timing: 1.5 h**

This step introduces P5 and P7 sequencing adapters, adds indexes, and amplifies the adapter tagged library.

22. Add the PCR mix.
  a. Pulse-spin the tube containing the Adaptase-tagged library.
  b. Add 30.3 μL of **indexing PCR mix** (see materials and equipment).
  c. If the optional pilot PCR in step 21 was performed, add 5.1 μL of nuclease-free water. **Otherwise, do not add water.**

***Note:*** The final PCR reaction volume is approximately 50.8 μL.

23. Amplify the library.
  a. Place the samples in a thermal cycler with the heated lid set to 105°C and run the following program:

**Table:** 

Temperature	Duration	Cycles
95°C	2 min	1
98°C	30 s	21
64°C	15 s	
72°C	30 s	
72°C	5 min	1
4°C	hold	–

***Note:*** If the optional pilot PCR was performed, use the cycle number determined from step 21. Otherwise, use 21 cycles as the default starting point. See troubleshooting, problems 1 and 5.

### Bead purification

**Timing: 20–40 min**

This step uses two rounds of AMPure cleanup to remove adapter dimers and residual primers from the final library.

24. Perform the first cleanup.
  a. Pulse-spin the PCR products.
  b. Add 40.6 μL AMPure beads (0.8× PCR product volume) to each tube.
  c. Mix thoroughly by gentle vortexing and pulse-spin to collect the liquid.
  d. Incubate for 5 min at 20°C–30°C.
  e. Place the tubes on a magnetic rack for about 5 min, or until the solution becomes clear.
  f. Carefully discard the supernatant.
  g. Wash the beads twice with 200 μL freshly prepared 80% ethanol per wash.
  h. Remove residual ethanol and air dry the beads at 20°C–30°C until no visible ethanol remains. Do not overdry the beads.
  i. Elute the DNA in 10–50 μL low EDTA TE Buffer.
25. Perform the second cleanup.
  a. Add AMPure beads at 0.8× the volume of the eluate from step 24.
  b. Repeat steps 24c-h.
  c. Elute the final libraries in 20–30 μL low EDTA TE Buffer.

**CRITICAL:** Ensure that the volume of AMPure beads is 0.8× the sample volume. Higher ratios may retain adapter dimers. See troubleshooting, problem 3.

**CRITICAL:** Do not overdry the beads, as this can significantly reduce DNA yield. See troubleshooting, problem 1.

### Concentration and fragment size check

**Timing: 1 h**

This step confirms library concentration and size distribution before sequencing.

26. Assess the final libraries.
  a. Measure the DNA concentration using Qubit. Expected concentration: 5–50 ng/μL.
  b. Evaluate the library size distribution using Bioanalyzer. Expected profile: a single broad peak spanning 300–500 bp, with no visible adapter dimers at approximately 120 bp (Figure S7). See troubleshooting, problem 3.
27. Store the final libraries at −20°C. Avoid repeated freeze thaw cycles.

### Sequencing

**Timing: Variable**

28. Prepare libraries for sequencing.
  a. Submit individual libraries for sequencing, or prepare a pooled library as required by the sequencing facility.
  b. If persistent dimer contamination is present and sufficient library material is available, perform gel size selection before sequencing:
    i. Combine the libraries into a pool and load the library on a 2% agarose gel.
    ii. Excise fragments approximately 400 bp–1.5 kb and recover the DNA using a gel extraction kit, for example, Qiagen QIAquick™ Gel Extraction Kit.
    iii. Re-quantify the recovered library before sequencing.
29. Sequence the libraries.
  a. Sequence the libraries on an Illumina NovaSeq X Plus using paired end 150 bp reads (2 × 150 bp).
  b. For routine applications, target approximately 10 Gb of raw sequencing data per library, corresponding to approximately 33 million read pairs for 2 × 150 bp sequencing.
  c. Demultiplex the sequencing output to generate paired R1 and R2 FASTQ files for each library.

**CRITICAL:** Assess the concentration and fragment size distribution of the library before sequencing. Libraries with substantial adapter dimer contamination require additional cleanup and reassessment, because short contaminating fragments can disproportionately occupy sequencing clusters and reduce usable read output.

***Note:*** The computational workflow processes paired-end low-input WGBS reads through trimming, alignment, deduplication, filtering, and methylation extraction. The automated workflow is implemented in MethylStar and is available at https://github.com/jlab-code/MethylStar.

***Note:*** Imputation is optional downstream procedure. The benchmark analyses described in quantification and statistical analysis use observed methylation fractions without imputation.

### Computational resource requirements

***Note:*** Computational requirements. The workflow requires a Unix-based environment. A multicore compute node with at least 32 GB RAM is recommended, including sufficient memory for the 20 GB sorting buffer used during methylation extraction. A workstation meeting these requirements can process individual libraries; HPC resources are preferable for processing multiple libraries concurrently.

***Note:*** Runtime. The approximate runtimes below refer to a single library and depend on sequencing depth, resource allocation, and system load. Processing from FASTQ files to methylation calls typically requires approximately 7–9 h under the configuration described here. Optional METHimpute processing requires approximately 5 additional hours.

***Note:*** Storage. The analysis directory occupies approximately 12 GB per library in this workflow. Approximately 15–20 GB of working disk space per library is recommended when intermediate files are retained. Concurrent processing requires correspondingly greater CPU, memory, and storage resources.

### Adapter trimming

**Timing: 20 min**

Trimming reduced the random priming bias in Read 1 and the Adaptase derived low complexity sequence bias at the beginning of Read 2 (Figures 1A and 1B).

30. Trim adapters and the 5′ ends with fastp.
  a. Remove adapters (step 1 of the two step trimming):
    fastp \
     -i “${sample}.R1.fq.gz” \
     -I “${sample}.R2.fq.gz” \
     -o “${sample}.R1.trimmed.fq.gz” \
     -O “${sample}.R2.trimmed.fq.gz” \
     --trim_poly_g \
     --qualified_quality_phred 20 \
     --detect_adapter_for_pe \
     -h “${sample}_fastp_adaptor.html” \
     -j “${sample}_fastp_adaptor.json”
  b. Trim 10 bp from the 5′ ends of both R1 and R2 reads (step 2):
    fastp \
     -i “${sample}.R1.trimmed.fq.gz” \
     -I “${sample}.R2.trimmed.fq.gz” \
     -o “${sample}.R1_trim10.fq.gz” \
     -O “${sample}.R2_trim10.fq.gz” \
     --trim_front1 10 \
     --trim_front2 10 \
     --disable_adapter_trimming \
     --length_required 30 \
     --cut_mean_quality 0 \
     -h “${sample}_fastp_trim10.html” \
     -j “${sample}_fastp_trim10.json”
    ***Note:*** We recommend double checking the quality of the trimmed reads, as well as the M-bias plot for the mapped BAM files, using FastQC and Bismark, as shown in the expected outcomes section. See troubleshooting, problems 2 and 4.

**Figure 1:**
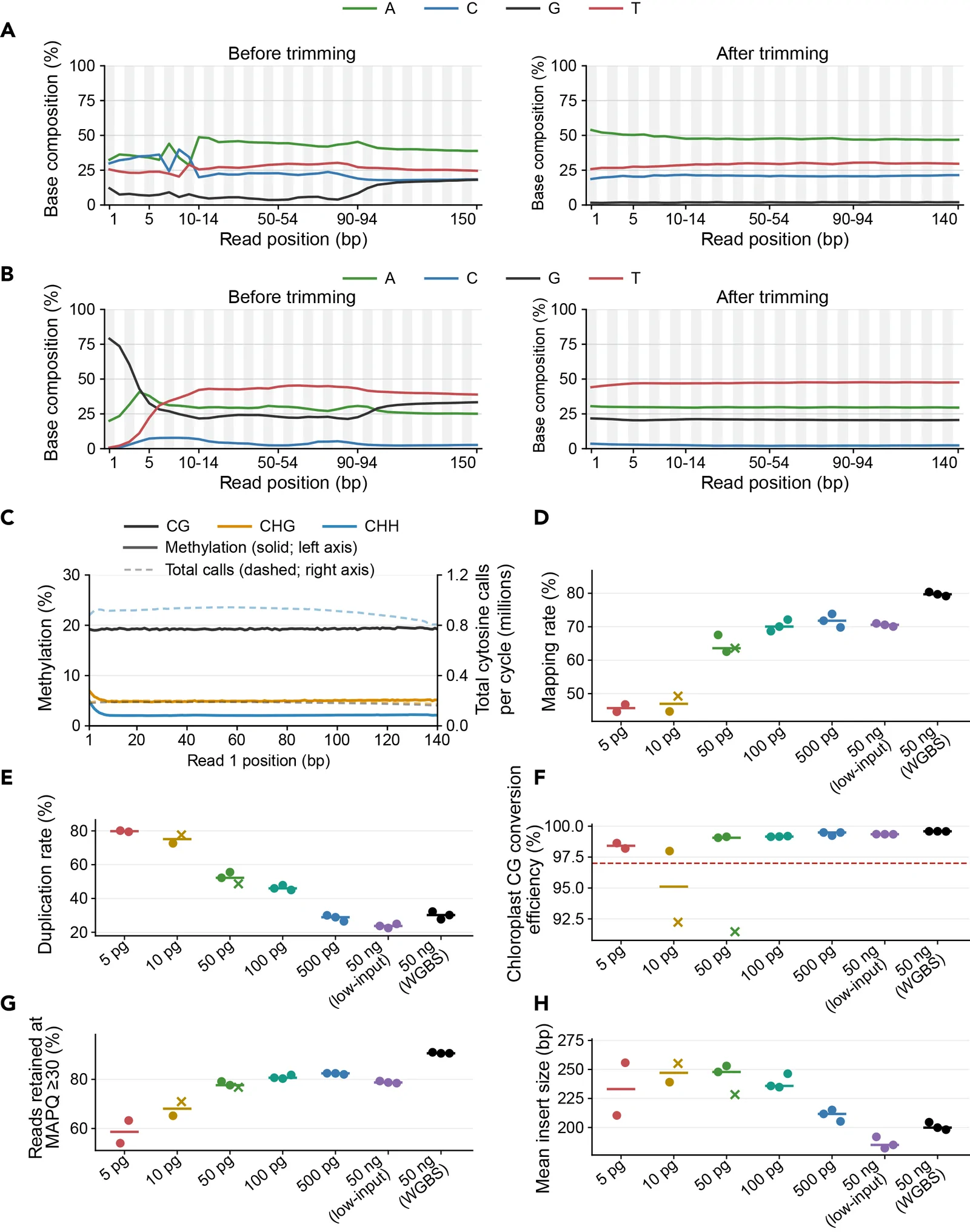
Quality control metrics for low-input and WGBS libraries across DNA inputs (A–C) Representative QC passed 50 pg library P50pg_1. (A and B) FastQC per base sequence content for Read 1 (A) and Read 2 (B) before (left) and after trimming (right), calculated from the complete FASTQ files without downsampling. (C) Read 1 M-bias plot generated from the filtered, deduplicated alignments, with overlapping mate calls suppressed during methylation extraction, showing methylation percentage (left axis) and total cytosine calls (right axis, millions) at each cycle for CG (black), CHG (orange), and CHH (blue). (D–H) Metrics for all 19 benchmark libraries: uniquely mapped read pairs as a percentage of pairs submitted to the aligner (D); duplicate pairs removed as a percentage of pairs entering deduplication (E); chloroplast CG conversion efficiency, with 97% indicated by the dashed line (F); read pair retention after MAPQ ≥ 30 and proper pair filtering relative to the post deduplication input (G); and mean insert size after MAPQ ≥ 30 filtering (H). Points denote individual libraries and horizontal bars indicate group medians. Crosses mark P10pg_3 and P50pg_3, which are included here and in the median calculations but excluded from Figures 2, 3, and S3–S6.

### Read mapping with Bismark

**Timing: 5 h**

This step aligns the trimmed reads to the reference genome in local mode to enhance mapping efficiency.^7^

31. Align the trimmed reads to the TAIR10 reference genome in local mode (--local):

bismark \

 -X 1000 \

 --non_directional \

 --local \

 --nucleotide_coverage \

 --genome “${genome_ref}” \

 --bowtie2 \

 -1 “${sample}.R1_trim10.fq.gz” \

 -2 “${sample}.R2_trim10.fq.gz” \

 -o “${output_dir}”

***Note:*** Bismark is specifically designed for bisulfite converted sequencing reads and accounts for the expected C to T and G to A conversions during alignment through in silico sequence conversion. Accordingly, the bisulfite aware alignment implemented by Bismark is sufficient to accommodate these conversion induced sequence differences without additional adjustment of mismatch parameters.

### Deduplication with Bismark

**Timing: 20 min**

This step removes PCR duplicates before downstream analysis.

32. Remove duplicate reads:

deduplicate_bismark \

 --paired-end \

 --bam \

 --output_dir “${output_dir}” \

 “${output_dir}/${sample}.R1_trim10_bismark_bt2_pe.bam”

### BAM sorting with SAMtools

**Timing: 20 min**

This step sorts the deduplicated alignments by coordinate.

33. Sort the deduplicated BAM file:

samtools sort \

 -@ 4 \

 -m 4G \

 -o “${sample}.dedup.sorted.bam” \

“${output_dir}/${sample}.R1_trim10_bismark_bt2_pe.deduplicated.bam”

### MAPQ30 filtering

**Timing: 30 min**

This step removes low quality and improperly paired alignments introduced by the post bisulfite adapter tagging (PBAT) approach.

34. Retain properly paired reads with MAPQ ≥ 30:

samtools view \

 -@ 4 \

 -h \

 -b \

 -f 0x2 \

 -F 0xF00 \

 -q 30 \

 -o “${sample}.dedup.sorted.mapq30.bam” \

 “${sample}.dedup.sorted.bam”

### Methylation extraction with Bismark

**Timing: 1 h**

This step extracts per cytosine methylation calls and generates genome wide cytosine reports.

35. Extract methylation calls for every cytosine from the aligned reads:

samtools sort -n -@ 4 -m 4G \

 -o “${sample}.dedup.sorted.mapq30.namesorted.bam” \

 “${sample}.dedup.sorted.mapq30.bam”

bismark_methylation_extractor \

--paired-end \

--buffer_size 20G \

--bedGraph \

--CX \

--genome_folder “${genome_ref}” \

-o “${output_dir}” \

“${sample}.dedup.sorted.mapq30.namesorted.bam”

36. Convert the Bismark coverage output into cytosine level files (CX reports):

coverage2cytosine \

--CX_context \

--coverage_threshold 1 \

--genome_folder “${genome_ref}” \

--dir “${output_dir}” \

-o “${sample}.covered_sites” \

“${output_dir}/${sample}.dedup.sorted.mapq30.namesorted.bismark.cov.gz”

### Imputing missing cytosines with METHimpute

**Timing: 5 h**

This step imputes cytosines not covered by sequencing using an HMM based binomial test for methylation status calling.

37. Run METHimpute with the CX reports from computational step 36 as input. The R code is provided in Methods S1.

## Expected outcomes

In this study, we serially diluted extracted *Arabidopsis* leaf DNA and used it as input starting from step 3 of the experimental section. Three independent library preparations were planned for each input group. One preparation each at 5 and 10 pg was unsuccessful during library preparation and was not repeated, leaving 19 benchmark libraries. All benchmark inputs were derived from the same extracted leaf DNA stock; the resulting libraries therefore represent technical library replicates rather than independent biological samples. Libraries with chloroplast bisulfite conversion efficiency below 97% in any of the CG, CHG, or CHH contexts failed conversion QC. P10pg_3 and P50pg_3 were excluded from Figures 2, 3, and S3–S6. All 19 benchmark libraries are included in Figures 1D–1H, S1, and S2, and Table S1; the 17 QC passed libraries are used in Figures 2, 3, and S3–S6.

**Figure 2:**
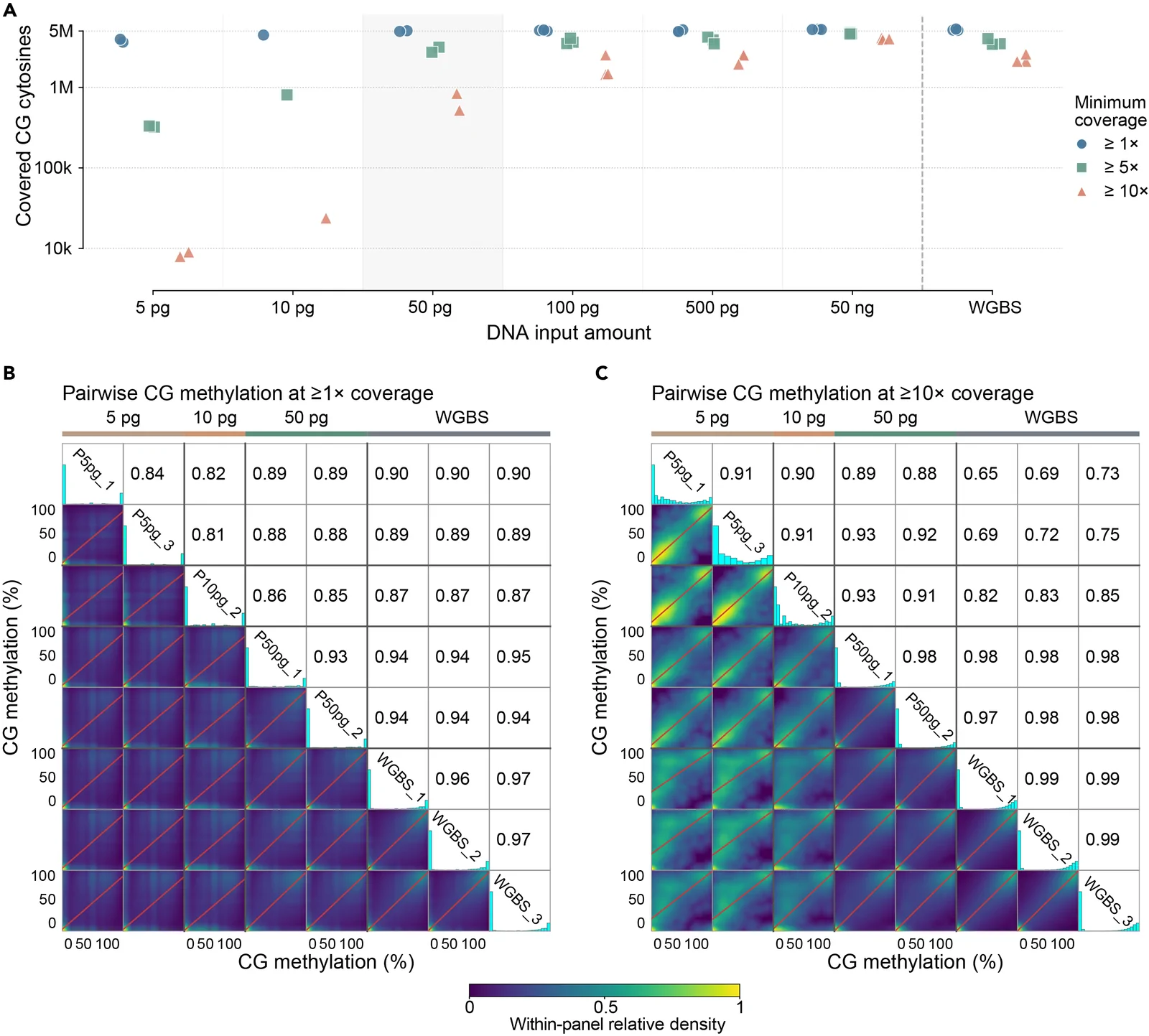
Nuclear CG recovery and pairwise methylation correlations in low-input and WGBS libraries (A) Numbers of nuclear CG cytosines covered at ≥ 1×, ≥ 5×, and ≥ 10× in each of the 17 QC passed libraries. Each point represents one library; the y-axis is logarithmic. (B and C) Pairwise Pearson correlations of methylation fractions at ≥ 1× (B) and ≥ 10× (C) for five QC passed low-input libraries (5 pg, n = 2; 10 pg, n = 1; 50 pg, n = 2) and three WGBS libraries. Off diagonal panels use strand resolved nuclear CG cytosines meeting the relevant threshold in both libraries. Upper triangles report Pearson’s r; lower triangles show relative density scaled within each panel, with linear fits in red (x, column library; y, row library). Diagonal histograms use each library’s own qualifying cytosines. Corresponding matrices for all 17 libraries are shown in Figures S4 and S5.

**Figure 3:**
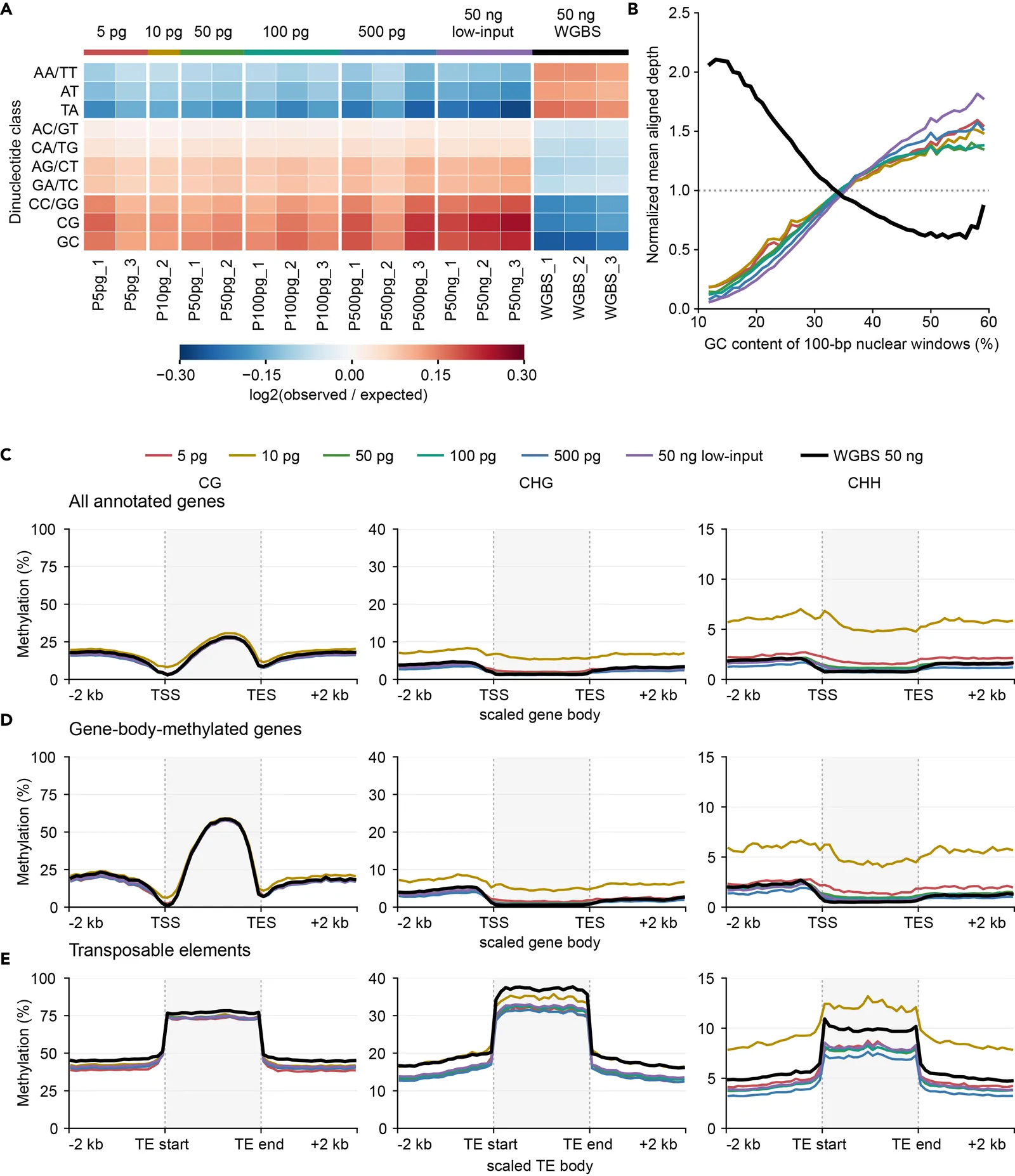
Sequence dependent coverage and genome wide cytosine methylation profiles across DNA inputs (A) Per library log_2_(observed/expected) coverage for ten reverse complement collapsed dinucleotide classes, calculated from all eligible nuclear alignments. Expected frequencies were derived from all adjacent ACGT only dinucleotides on nuclear chromosomes 1–5, including positions with zero aligned depth; observed frequencies weighted each occurrence by the mean aligned depth of its two bases. (B) Normalized coverage of 100 bp nuclear windows across GC content classes. QC passed libraries were pooled within each input group, and 1,000,000 complete read pairs were randomly sampled across all reference sequences before coverage was restricted to nuclear chromosomes. Mean coverage in each GC class was divided by the mean across all eligible nuclear windows; only classes containing ≥ 1,000 reference windows are shown. (C–E) CG, CHG, and CHH methylation profiles across annotated nuclear genes (C), the set of 5,314 gbM genes (D), and TEs (E), each with 2 kb flanks. Curves are unweighted means of observed library level profiles. Numbers of QC passed libraries were 2, 1, 2, 3, 3, 3, and 3 for 5 pg, 10 pg, 50 pg, 100 pg, 500 pg, low-input 50 ng, and WGBS 50 ng, respectively.

The following results describe the expected quality metrics and sequencing characteristics obtained with our low-input protocol across the tested input range (5 pg to 50 ng), compared with standard WGBS at 50 ng. Alignment metrics were derived from the alignment and deduplication reports and BAM files. For downstream analyses, such as sequence context and GC content biases, the results were computed from filtered BAM files with MAPQ ≥ 30. Finally, our low-input protocol showed better library metrics and CG site recovery at 50 pg than at 5–10 pg, and to obtain a more confident result, we recommend starting with at least 50 pg of starting material.

### Library quality and alignment statistics

Figures 1A–1C show the QC passed library P50pg_1 (50 pg). Trimming reduced the random priming bias in Read 1 and the Adaptase derived low complexity sequence bias at the beginning of Read 2 (Figures 1A and 1B). M-bias analysis showed residual position dependent methylation bias in the first few cycles of Read 1, particularly in the CHG and CHH contexts (Figure 1C). For analyses focused on CHG or CHH methylation, additional 5′ trimming of Read 1 may be considered, with the trim length selected from the dataset specific M-bias profile. The effect of such additional trimming was not evaluated in this benchmark and should be assessed against the associated loss of usable sequence and coverage.

Mapping efficiency was consistently high for WGBS (79.2%–80.4%) and remained stable across mid to high low-input concentrations (62.5%–73.8% at 50 pg–50 ng; Figure 1D). The reduced mapping efficiency of the low-input protocol compared to WGBS reflects the PBAT library architecture, which uses random priming of bisulfite converted single stranded DNA, introducing primer bias and higher rates of non aligning reads as reported for PBAT based libraries.^8^^,^^9^^,^^10^^,^^11^ The lower mapping rate at 5–10 pg (44.6%–49.3% at 5–10 pg; Figure 1D) is further explained by the high proportion of adapter dimers when library complexity is severely limited.

As expected, the duplication rate increased inversely with input amount, from ∼30% at WGBS 50 ng to ∼80% at low-input 5 pg, reflecting the reduced library complexity at lower starting material (Figure 1E). Among QC passed libraries, chloroplast bisulfite conversion efficiency exceeded 99.0% in all three sequence contexts at inputs of 50 pg–50 ng and in WGBS controls, and ranged from 98.0% to 98.7% at 5–10 pg **(**Figure 1F; Figures S1 and S2). The remaining QC passed 10 pg library, P10pg_2, showed reduced conversion efficiency in the non CG contexts and was therefore interpreted cautiously in downstream methylation profile analyses.

After retaining only properly paired read pairs with MAPQ ≥ 30 (MAPQ30), which excludes multi mapping and low quality alignments, low-input libraries at mid to high concentrations showed consistent retention rates of ∼80%, relative to the post deduplication input (Figure 1G). Standard WGBS retained ∼90% of read pairs after MAPQ30 filtering, consistent with the higher mapping specificity of standard WGBS directional libraries (Figure 1G).

Moreover, mean insert sizes from MAPQ30 filtered BAM files ranged from 182–192 bp (low-input 50 ng) to 210–256 bp (low-input 5 pg), with the overall size falling in the range of 182–256 bp (Figure 1H). Standard WGBS showed a mean insert size with lower variability than low-input, with a range of 198–205 bp, which is expected for WGBS libraries prepared from sonicated genomic DNA (Figure 1H). Interestingly, the low-input protocol showed a trend of increasing insert size at lower input concentrations, which may reflect preferential amplification of larger unique molecules when library complexity is limited. Notably, the low-input 5 pg group showed the widest variation in insert size (210–256 bp), indicating increased stochastic variability at extreme low-input. All groups produced insert sizes within the expected range for PE150 sequencing, confirming that the low-input libraries are structurally compatible with standard Illumina short read sequencing (Figure 1H). Complete per replicate metrics are provided in Table S1.

### Input-dependent CG detection and methylation concordance

At 1× minimum coverage, low-input libraries at ≥ 50 pg captured 4.92–5.26 million nuclear CG cytosines per library (Figure 2A). At lower inputs, these counts declined to 4.46 million (10 pg) and 3.64–3.95 million (5 pg). Applying a more stringent 5× threshold sharpened this distinction (Figure 2A). At extremely low-input levels (5–10 pg), the number of covered CG cytosines decreased to 0.32–0.81 million per library (Figure 2A). In contrast, low-input libraries at 50 pg retained 2.72–3.15 million CG cytosines at ≥ 5× coverage (Figure 2A). Figure S3 shows the full depth overlap of nuclear CG cytosines between each low-input group and WGBS at ≥ 1× and ≥ 5× coverage. Cytosines were filtered independently within each QC passed library before the qualifying site sets were combined by union within each group; each low-input union was compared with the same union of the three WGBS libraries. These comparisons describe cumulative site recovery at the available sequencing depths and group sizes. Consistent with this coverage landscape, pairwise Pearson correlations of per CG methylation levels at a ≥ 1× minimum coverage threshold were high across all samples (Figure 2B; Figure S4). Within group correlations were r = 0.930 for the two 50 pg libraries and r = 0.965–0.968 among WGBS replicates, while cross protocol correlations between low-input libraries prepared from ≥ 50 pg and WGBS ranged from r = 0.940–0.967, declining modestly at extremely low-input (r = 0.866–0.900 at 5–10 pg; Figure 2B; Figure S4). At a ≥ 10× minimum coverage threshold, correlations were calculated on the cytosines independently meeting this threshold in both libraries of each pair (Figure 2C; Figure S5). Cross protocol correlations between low-input libraries prepared from ≥ 50 pg and WGBS remained high (r = 0.974–0.988, mean = 0.983), whereas low-input libraries prepared from 5–10 pg showed reduced concordance with WGBS (r = 0.651–0.854, mean = 0.748; Figure 2C; Figure S5), with only 3,806–12,410 jointly covered nuclear CG cytosines in these comparisons. At ≥ 10×, the two 5 pg libraries retained only 7,950–9,050 CG cytosines individually, compared with 520,577–840,390 in the two QC passed 50 pg libraries.

### Sequence context bias

For Figure 3A, each QC passed library was analyzed independently using all eligible nuclear alignments. For Figure 3B only, eligible complete read pairs were pooled within each input group, and 1 million pairs were randomly sampled without replacement before restricting the coverage analysis to nuclear chromosomes 1–5.

Dinucleotide coverage bias profiles were highly consistent across all six low-input concentrations, with observed/expected ratios ranging from 0.83 to 1.19 (Figure 3A). Low-input libraries showed relative depletion of AA/TT, AT, and TA and enrichment of CC/GG, CG, and GC. In contrast, the WGBS library showed enrichment of AA/TT, AT, and TA and depletion of CC/GG, CG, and GC relative to the nuclear reference expectation (Figure 3A).

#### GC-dependent coverage bias

WGBS showed higher normalized coverage in lower GC windows and lower coverage in higher GC windows over the displayed range (Figure 3B). In contrast, low-input libraries generally showed increasing normalized coverage with GC content over the displayed range. Only GC classes represented by at least 1,000 reference windows are displayed (12–59% GC), accounting for 99.6% of eligible windows.

The low-input libraries shared the same overall direction of dinucleotide and GC dependent coverage bias across the tested input amounts (Figures 3A and 3B). Per library GC dependent coverage profiles without pooling or downsampling are provided in Figure S6. Coverage is normalized within each library using all valid nuclear windows; GC classes are shown when represented by at least 100 windows. We also compared methylation patterns and levels between low-input and WGBS libraries. Figures 3C–3E show profiles for annotated nuclear genes, a predefined set of 5,314 gene body methylated (gbM) genes, and nuclear transposable elements (TEs), respectively, in CG, CHG, and CHH contexts across scaled feature bodies with 2 kb upstream and downstream flanks. As expected, both protocols showed broadly consistent methylation patterns across contexts and annotations, although the single QC passed 10 pg library retained elevated non CG methylation, which may reflect incomplete bisulfite conversion (Figures 3C–3E). For TEs, most low-input groups showed lower methylation levels than WGBS, particularly in CHG and CHH contexts, consistent with previous observations that WGBS can overestimate non CG methylation levels (Figure 3E).^12^

#### Application to individual SAMs

We prepared five libraries from independently dissected SAMs, using one SAM per library. After trimming and Bismark alignment, the libraries yielded 15,896,654–27,171,808 unique best hit read pairs before deduplication. After deduplication and retention of properly paired alignments with MAPQ ≥ 30, 8,977,907–12,991,186 read pairs remained, corresponding to mean depths of 20.10×–28.99× and genome coverage of 95.65%–96.54%. The libraries detected 5,174,626–5,274,207 nuclear CG cytosines at ≥ 1× coverage and 3,910,357–4,592,907 at ≥ 5× coverage. Bisulfite conversion, estimated from endogenous chloroplast cytosines, ranged from 99.49% to 99.54% in the CG context, 99.48% to 99.53% in CHG, and 99.43% to 99.49% in CHH. Per library sequencing, coverage, and conversion metrics are provided in Table S2. These SAM libraries form a separate validation dataset and were not included in the leaf DNA dilution benchmark.

## Quantification and statistical analysis

Nuclear CG cytosines were filtered in R using the data.table package, retaining sites with m + u ≥ t (t = 1, 5, or 10). Sites were identified by chromosome, 1 based coordinate, and strand; complementary CG positions were not collapsed. At t = 1 or 10, pairwise Pearson correlations (stats::cor, method = “pearson”) were calculated from m/(m + u) at sites meeting the threshold in both libraries, with equal site weights and no imputation. Choose the minimum coverage according to the analysis objective and the number of sites retained. A ≥ 1× threshold is useful for assessing detection breadth, but includes sites supported by only one call, for which the observed methylation fraction can only be 0 or 1. For exploratory site level comparisons in this dataset, ≥ 5× can be considered a starting filter, with suitability assessed from the retained site count and the downstream analysis rather than treated as a universal requirement. A ≥ 10× threshold provides greater read support per retained site but can substantially reduce site recovery, particularly in 5–10 pg libraries. For pairwise comparisons, require the threshold in both libraries and report the number of jointly covered sites.

For Figure S3, sites were filtered independently in each QC passed library at ≥ 1× or ≥ 5× and unioned within input group without pooling counts before thresholding. Each low-input union was compared with the three library WGBS union. Euler diagrams were fitted with eulerr v7.0.4 using a common scale. For Figures 2B, 2C, S4, and S5, off diagonal panels used pair specific shared sites; colors show panel scaled relative density, red lines show linear fits, and diagonal histograms use each library’s own qualifying sites. No subsampling or imputation was applied.

For Figure 3B, QC passed libraries were pooled within the input group, and 1,000,000 complete read pairs were randomly sampled across all reference sequences before restricting coverage to 100 bp nuclear windows. GC class coverage was normalized to the mean across all eligible nuclear windows. Metaprofiles in Figures 3C–3E were generated with methimpute::plotEnrichment v1.28.0 using 20 scaled feature body bins and twenty 100 bp bins per 2 kb flank. Covered site methylation fractions (m + u ≥ 1) were averaged within libraries; group curves are unweighted means of library level profiles without pooling, downsampling, or imputation.

## Limitations

Inputs below 50 pg, particularly 5–10 pg, can still generate libraries but show reduced library fidelity, lower CG recovery, and weaker downstream methylation concordance; therefore, 50 pg is recommended as the minimum input for robust methylome profiling.

Due to the trace amounts of starting material, an increased number of PCR enrichment cycles (typically 19–21 cycles) is required to achieve the minimum library concentration for sequencing. However, higher cycle numbers inevitably lead to an elevated duplication rate and a concomitant reduction in library complexity. Researchers must strike a balance between achieving sufficient library yield and maintaining high data utilization; we recommend using the minimum number of cycles necessary to reach the sequencing threshold.

## Troubleshooting

### Problem 1

Low library yield, related to steps 1, 2, 17 and 21–25.

### Potential solution

• Ensure that cryo milling fully disrupts the tissue and that tissue lysis is sufficiently thorough.
• Avoid excessive incubation times during the bisulfite treatment; prolonged exposure to conversion reagents can lead to severe DNA fragmentation and degradation, reducing the template quality for library construction.
• Perform a pilot PCR to determine the optimal cycle number and avoid insufficient amplification:
  ○ Take a one quarter aliquot of the Adaptase tagged library, as described in step 21, before the main indexing PCR.
  ○ Amplify the pilot reaction for 21 cycles, purify the product, measure its concentration, and record the pilot elution volume.
  ○ Estimate the total pilot yield by multiplying the measured concentration by the pilot elution volume.
  ○ Use the pilot total yield as an empirical guide for selecting the cycle number for the remaining 3/4 template, accounting for its approximately 3 fold greater template input and the planned final elution volume.
  ○ Increase the cycle number if the estimated yield is insufficient, or decrease it to minimize the duplication rate.
• Avoid over drying the magnetic beads during the cleanup step, as this significantly reduces the DNA yield obtained during elution.

### Problem 2

M-bias artifacts or distortions, related to computational step 30.

### Potential solution

• Check whether the first 10 bp were correctly trimmed from the 5′ ends of both R1 and R2 reads.
• Inspect FastQC and Bismark M-bias plots after trimming.
• If residual end specific bias remains, trim a few additional bases from the affected end and reassess the M-bias plots. Use the minimum additional trimming required, because excessive trimming shortens the usable reads and may reduce mapping efficiency and effective coverage.

### Problem 3

Adapter contamination or visible adapter dimers, related to steps 24–26 and 28.

### Potential solution

• Assess adapter dimer contamination by molar fraction rather than mass, as dimers may appear minor on the Bioanalyzer trace but represent a substantial fraction of library molecules.
• Small amounts of adapter dimers are usually acceptable for sequencing, but high levels can reduce the number of usable reads.
• For both cleanup steps 24 and 25, ensure that AMPure beads are used at 0.8× the corresponding sample volume. Ratios above 0.8× may retain more adapter dimers.
• If dimer contamination remains and the library concentration is sufficient, remove fragments below 400 bp by gel size selection and gel extraction. Use this option cautiously, as it can reduce library yield and increase the risk of contamination.

### Problem 4

Low mapping rate, related to computational steps 30 and 31.

### Potential solution

• Confirm that adapter trimming and 10 bp 5′ trimming were performed correctly.
• Confirm that Bismark alignment was run with --non_directional and --local, as specified in step 31.
• Note that local alignment may increase M-bias or introduce alignment related bias.

### Problem 5

High duplication rate and low library complexity, related to step 23 and 32.

### Potential solution

• Use the minimum number of PCR enrichment cycles necessary to reach the sequencing threshold; higher cycle numbers increase the duplication rate and reduce library complexity.
• Determine the optimal cycle number with a pilot PCR (see problem 1) rather than defaulting to a high cycle number.
• Where possible, start from at least 50 pg of input DNA, because lower inputs (5–10 pg) have reduced library complexity and therefore higher duplication rates.

## Resource availability

### Lead contact

Further information and requests for resources and reagents should be directed to and will be fulfilled by the lead contact, Frank Johannes (frank@johanneslab.org).

### Technical contact

Technical questions on executing this protocol should be directed to and will be answered by the technical contact, Frank Johannes (frank@johanneslab.org).

### Materials availability

This protocol did not generate new unique reagents. All oligonucleotides used, including the custom P5L and P7L indexing primers and the P5L random primer, are listed with their sequences in the materials and equipment section and are available from the lead contact upon request.

### Data and code availability

• The accession number for the raw sequencing data reported in this paper is GEO: GSE338396. Per-library alignment metrics for the leaf DNA benchmark are provided in Table S1, and per-library validation metrics for individual SAMs are provided in Table S2.
• The analysis pipeline used for read trimming, alignment, filtering, deduplication, methylation calling, and imputation is available at https://github.com/jlab-code/MethylStar. An archived version of MethylStar v.1.5 is available on Zenodo at https://doi.org/10.5281/zenodo.23082324. The R code used to generate input files for METHimpute is provided in Methods S1.
• Any additional information required to reanalyze the data reported in this protocol is available from the lead contact upon request.

## Acknowledgments

This work was partly funded by the 10.13039/501100002347Bundesministerium für Bildung und Forschung through the epiSOMA project. J.Y. was supported by the 10.13039/501100004543China Scholarship Council (CSC) (fellowship no. 202208440069). We thank Claude Becker for providing hands-on training in WGBS library preparation to our laboratory, which helped establish the experimental foundation of this protocol. We are grateful to Christine Wurmser for her expert advice and technical expertise during optimization of the library preparation workflow.

## Author contributions

J.Y. established and optimized the low-input WGBS library preparation and data analysis workflows, performed the benchmarking evaluation, and prepared the manuscript. B.T.V. performed conventional WGBS library preparation and benchmarking analysis, contributed to analysis workflow optimization, MethylStar integration, and manuscript preparation. F.J. supervised the project and contributed to troubleshooting.

## Declaration of interests

The authors declare no competing interests.

## Declaration of generative AI and AI-assisted technologies in the manuscript preparation process

During the preparation of this work, the authors used ChatGPT for language editing and code optimization. The authors reviewed and edited the output as needed and take full responsibility for the content of the published article.

## Supplemental information

Document S1. Figures S1–S7

Table S1. Per library alignment metrics for the benchmark libraries, related to Expected outcomes

Table S2. Per library sequencing, alignment, coverage, and conversion control metrics for the individual SAM validation, related to Before you begin and Expected outcomes

Methods S1. R code for generating METHimpute files, related to the “Imputing missing cytosines with METHimpute” step 37
