# Supplementary material for: Protocol for low-input whole-genome bisulfite sequencing in Arabidopsis
Source: STAR Protoc. 2026 Oct 30;7(4):104928. doi: 10.1016/j.xpro.2026.104928 (PMC13744330; doi:10.1016/j.xpro.2026.104928)
Supplement: Supplementary file 1 [file mmc1.pdf]

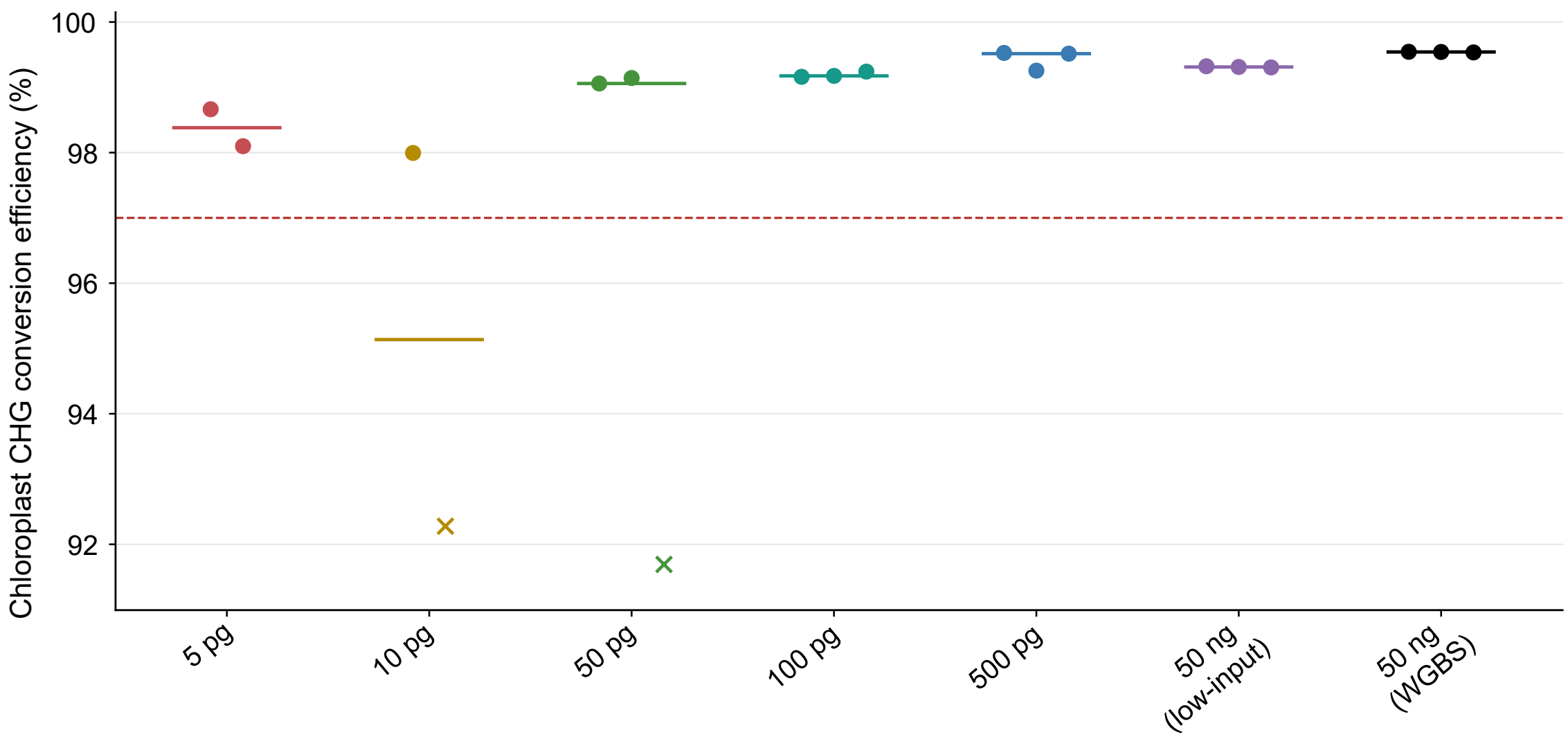

Figure S1. Chloroplast bisulfite conversion efficiency in the CHG context, related to Expected outcomes.
All 19 benchmark libraries are shown: 5 pg (n = 2), 10 pg (n = 2), 50 pg (n = 3), 100 pg (n = 3), 500 pg (n = 3), low input 50 ng (n = 3), and WGBS 50 ng (n = 3). Each point represents a separately prepared technical library replicate. Crosses mark P10pg_3 and P50pg_3, which did not meet the prespecified QC criterion requiring ≥ 97% conversion in each of the three contexts (CG, CHG, and CHH) but are retained for QC display. The dashed line marks 97%.

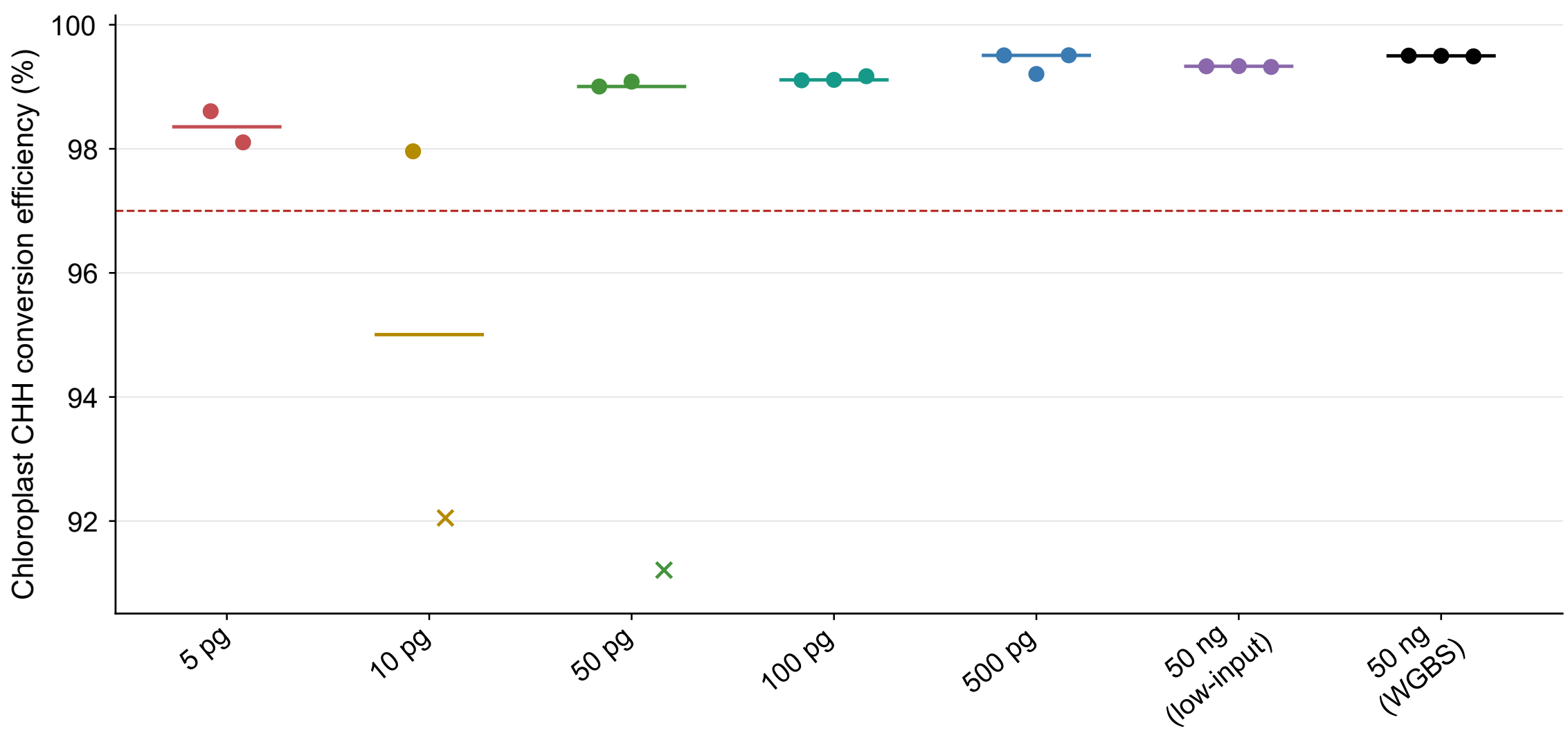

Figure S2. Chloroplast bisulfite conversion efficiency in the CHH context, related to Expected outcomes.
The same 19 libraries and group sizes as in Figure S1 are shown. QC symbols are as in Figure S1; the dashed line marks 97%.

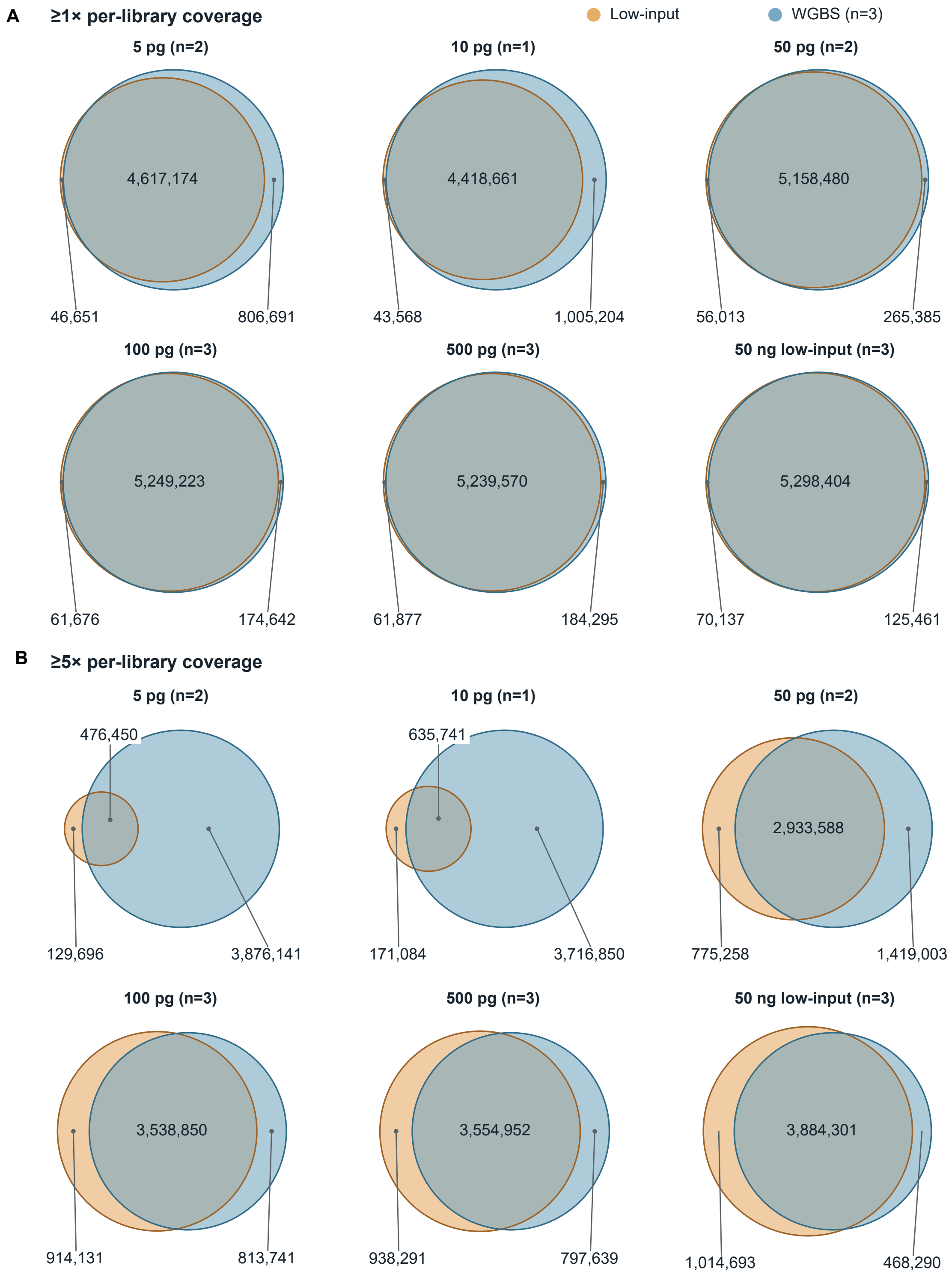

Figure S3. Full depth overlap of nuclear CG cytosines recovered by low input and WGBS libraries, related to Figure 2A.

Strand resolved nuclear CG cytosines on chromosomes 1–5 were selected independently in each QC passed library at ≥ 1× (A) or ≥ 5× (B) coverage and then unioned within each input group; a site was included if at least one library independently met the threshold. Counts were not pooled before thresholding, and complementary CG positions were retained separately. Low input group sizes were 5 pg (n = 2), 10 pg (n = 1), 50 pg (n = 2), 100 pg (n = 3), 500 pg (n = 3), and 50 ng (n = 3). Each low input union was compared with the same three library WGBS union (WGBS_1–3; source IDs S45_1–3). Orange and blue denote low input and WGBS unions, respectively; labels report exact low input only, shared, and WGBS only counts. All area proportional Euler diagrams use a common scale. These unions reflect the available sequencing depths and group sizes.

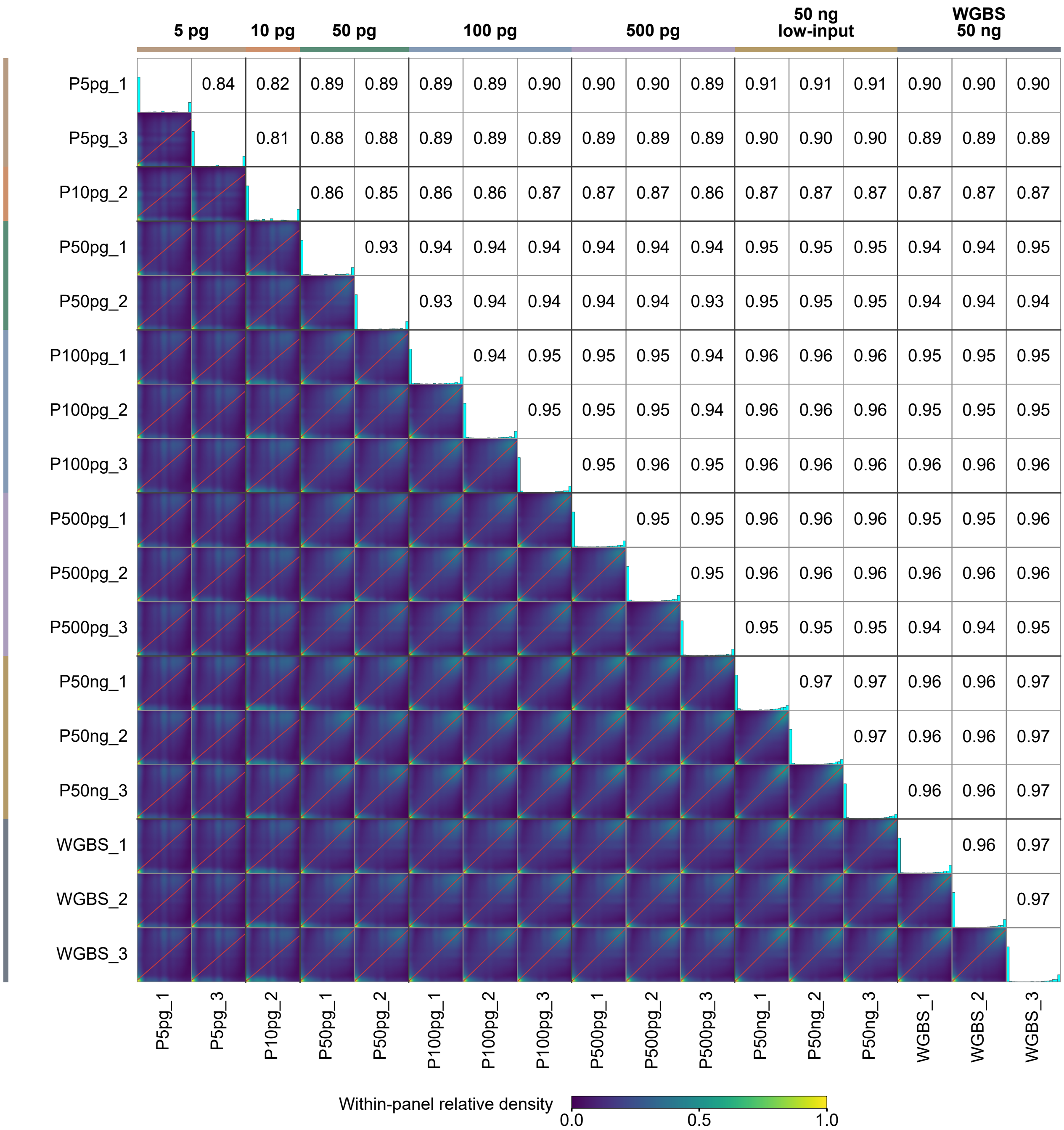

Figure S4. Pairwise nuclear CG methylation correlations among the 17 QC passed libraries at ≥ 1× coverage, related to Figure 2B.

Libraries are ordered as 5 pg (n = 2), 10 pg (n = 1), 50 pg (n = 2), 100 pg (n = 3), 500 pg (n = 3), low input 50 ng (n = 3), and WGBS 50 ng (n = 3). WGBS_1–3 correspond to source libraries S45_1–3. For each pair, Pearson's r was calculated from methylation fractions at strand resolved nuclear CG cytosines meeting the threshold in both libraries. Upper triangles report r rounded to two decimal places; lower triangles show relative density scaled within each panel, with linear fits in red (x, column library; y, row library); diagonal histograms show each library's own qualifying cytosines.

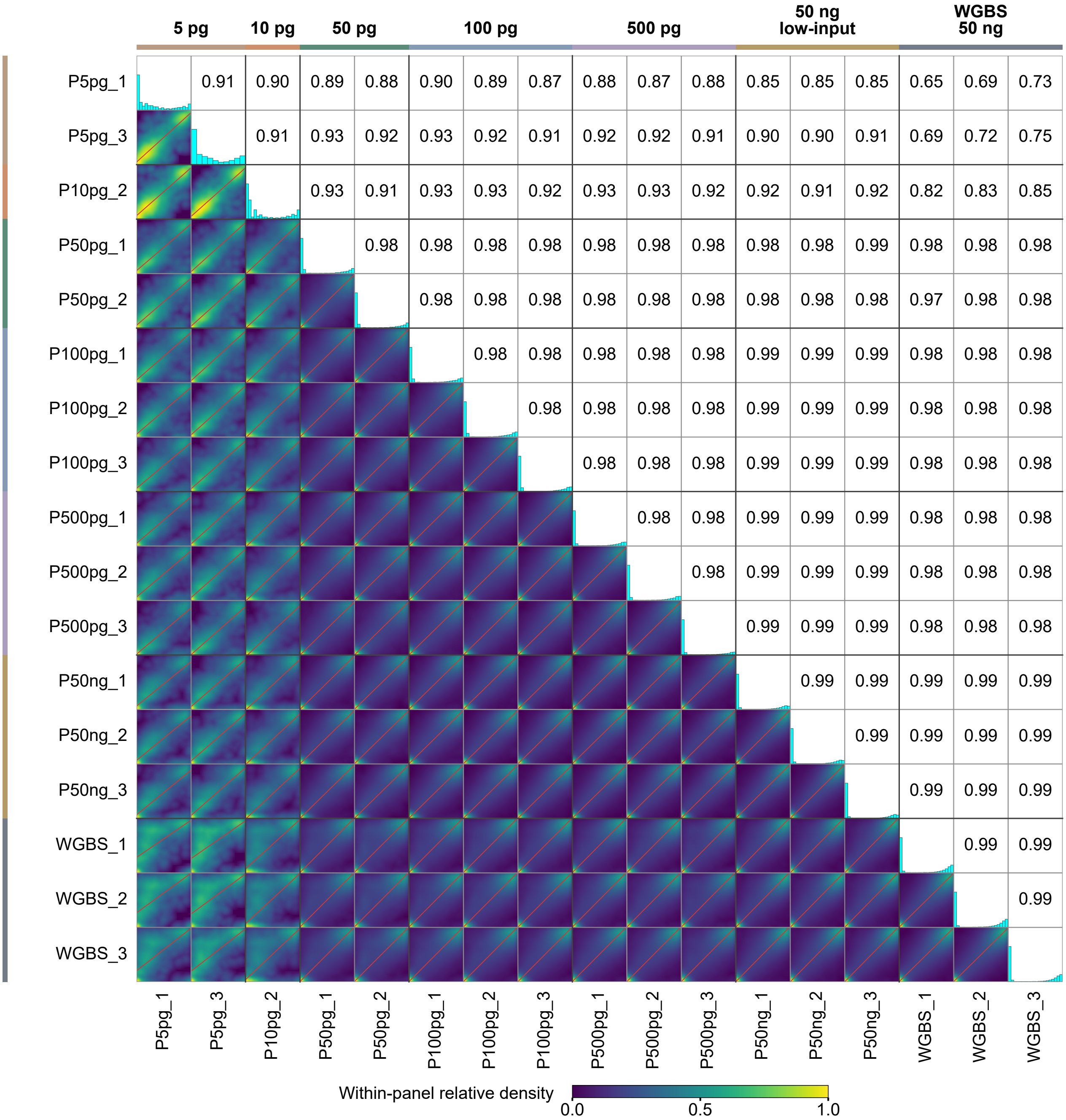

Figure S5. Pairwise nuclear CG methylation correlations among the 17 QC passed libraries at ≥ 10× coverage, related to Figure 2C.

Library order and display conventions are as in Figure S4. Each off diagonal comparison uses strand resolved nuclear CG cytosines meeting the ≥ 10× threshold in both libraries; diagonal histograms use each library’s own qualifying cytosines. Qualifying site sets therefore differ among library pairs and from those used in Figure S4.

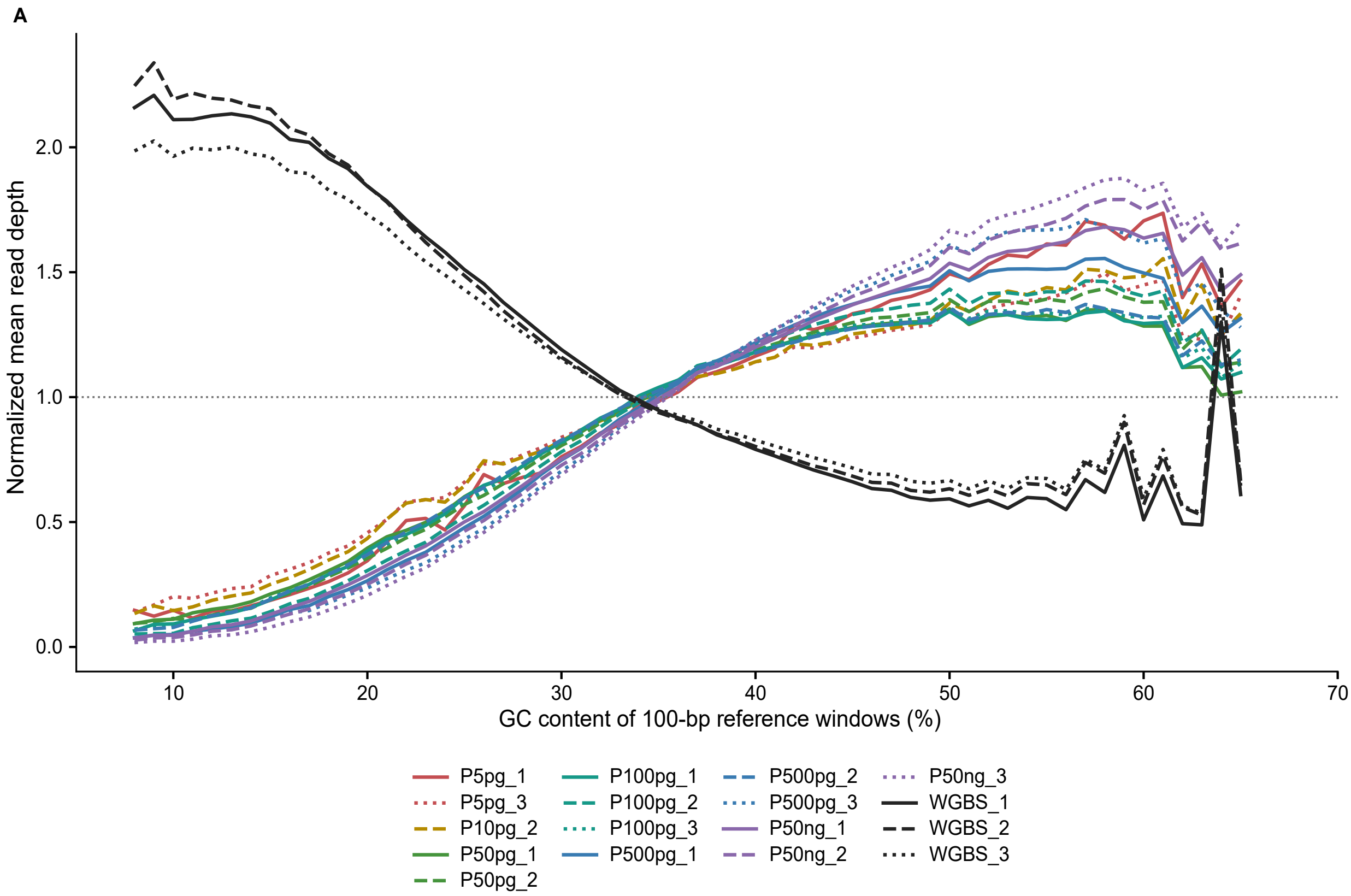

Figure S6. GC dependent coverage in each of the 17 QC passed libraries, related to Figure 3B.
Libraries were analyzed separately using all eligible nuclear alignments. Nuclear chromosomes 1–5 were divided into non overlapping, ACGT only 100 bp windows. Mean depth in each integer GC content class was divided by the mean across all valid windows, including classes omitted from display. Only classes containing ≥ 100 windows are shown. Colors denote input groups and line styles identify libraries. The dotted line marks normalized coverage of 1.

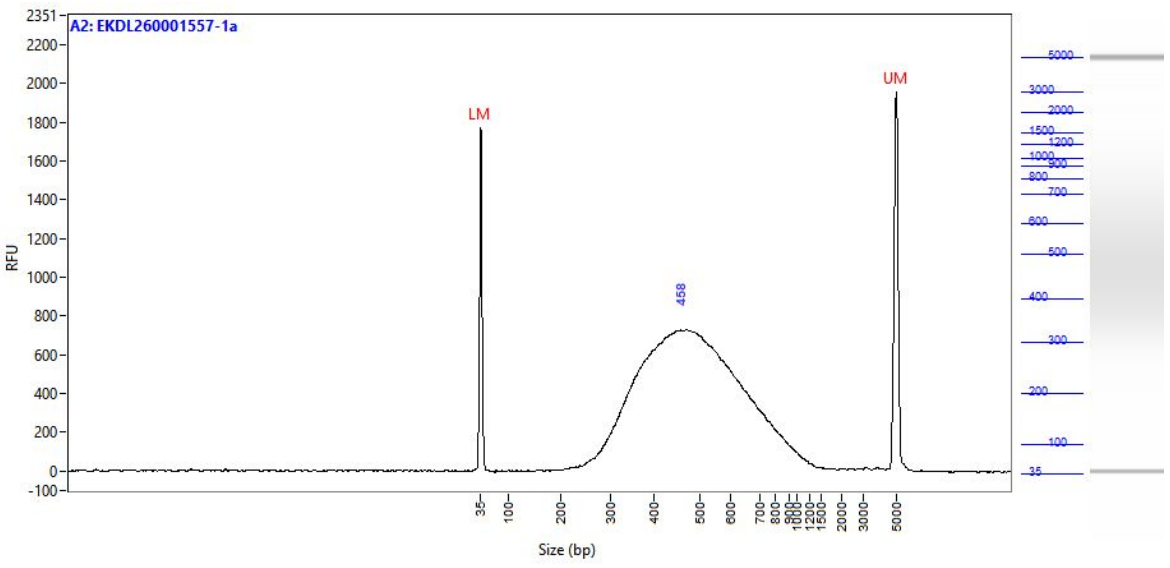

Figure S7. Example Bioanalyzer electropherogram illustrating library fragment size quality control, related to "Concentration and fragment size check."
The trace shows a library prepared using this protocol and is provided as a qualitative example of an acceptable fragment size distribution. LM and UM indicate the lower and upper markers, respectively.

Additional supplemental files

Table S1. Per library alignment metrics for the benchmark libraries, related to Expected outcomes.
Excel file.

Table S2. Per library sequencing, alignment, coverage, and conversion control metrics for the individual SAM validation, related to Before you begin and Expected outcomes.
Excel file.

File S1. R code for generating METHimpute files, related to the "Imputing missing cytosines with METHimpute" step.
R script.
